# Dietary Matcha Attenuates High-Fat High-Sugar Diet-Induced Cardiomyocyte Hypertrophy in Association with Gut Microbiota Remodeling in Mice

**DOI:** 10.3390/nu18182961

**Published:** 2026-09-09

**Authors:** Gopal Lamichhane, Jong-Beom Jin, Jiaqing Guo, Da-Yeon Lee, Femi Olawale, Cody Moss, Guolong Zhang, Jiyoung Bae, Edralin A. Lucas, Yoo Kim

**Affiliations:** 1 Department of Nutritional Sciences, Oklahoma State University, Stillwater, OK 74078, USA; gopal.lamichhane@okstate.edu (G.L.); jojin@okstate.edu (J.-B.J.); dayeon.lee@okstate.edu (D.-Y.L.); femi.olawale@okstate.edu (F.O.); cody.moss10@okstate.edu (C.M.); jiyoung.bae@okstate.edu (J.B.); edralin.a.lucas@okstate.edu (E.A.L.); 2 Department of Animal and Food Sciences, Oklahoma State University, Stillwater, OK 74078, USA; jiaqing.guo@okstate.edu (J.G.); glenn.zhang@okstate.edu (G.Z.)

**Keywords:** cardiac hypertrophy, matcha, gut–heart axis

## Abstract

**Objectives**: Obesity is associated with cardiac remodeling characterized by cardiomyocyte hypertrophy and fibrosis, eventually leading to heart failure. This study aimed to evaluate whether dietary matcha supplementation could be effective in reducing high-fat, high-sugar diet (HFD)-induced cardiac remodeling in mice, and whether this effect is linked to changes in the gut microbiota along the gut–heart axis. **Methods**: Six-week-old C57BL/6J mice were randomly divided into normal chow diet (NCD) or HFD (without matcha, 0.2% matcha, or 1% matcha) groups. Dietary intervention continued for 10 weeks with continuous monitoring of body weight. After completion of the intervention, hearts were collected, weighed, and processed for histological examination of cardiomyocyte size with wheat germ agglutinin (WGA) staining and collagen deposition with Picrosirius Red staining. The gut microbiome was evaluated using 16S rRNA sequencing of gDNA from feces, and correlation analysis was performed between microbial taxa and cardiometabolic parameters. **Results**: After 10 weeks of dietary intervention, heart weight was significantly lower in mice treated with 1% matcha than in HFD controls. WGA staining showed a significant increase in cardiomyocyte size in the HFD group compared with the NCD group, whereas both the 0.2% and 1% matcha-supplemented groups revealed significantly smaller cardiomyocytes than the HFD controls. In addition, both matcha doses significantly reduced cardiac collagen deposition relative to HFD controls. In the gut, matcha supplementation displayed a marked increase in the abundance of beneficial gut microbiota, including *Ligilactobacillus*, *Lactobacillus*, and *Oscillospiraceae*, while reducing pathogenic *Enterobacteriaceae* and *Klebsiella*, particularly at the high dose. **Conclusions**: Dietary matcha supplementation attenuated HFD-induced cardiac hypertrophy and fibrosis in mice, independently of body weight, in association with modulation of the gut microbiota along the gut–heart axis. Future studies should determine whether the observed reductions in cardiac hypertrophy and fibrosis translate into measurable improvements in cardiac function and whether the associated gut microbiota alterations exert causal effects on cardiac remodeling.

## 1. Introduction

Cardiovascular diseases (CVDs), including ischemic heart disease and stroke, are the leading causes of morbidity and mortality worldwide [1]. These conditions arise from structural and functional abnormalities in the heart and blood vessels, often progressing to heart failure—a state in which the heart is unable to pump sufficient blood to meet the body’s metabolic demands [2,3,4]. One of the hallmarks that precedes heart failure is cardiac hypertrophy, a compensatory response of the heart to increased workload or chronic stressors such as obesity and hypertension [5]. Cardiac hypertrophy is primarily characterized by an increase in cardiomyocyte size and thickening of the ventricular walls. In the early stages, this growth serves as an adaptive mechanism to preserve cardiac output. However, under sustained or excessive stress, the initially beneficial hypertrophy becomes maladaptive, leading to ventricular dilation, diminished contractile function, and ultimately, heart failure [5,6].

Among the modifiable risk factors contributing to pathological cardiac remodeling, obesity has emerged as a key driver of cardiac disease progression [7,8,9]. Beyond its association with traditional cardiometabolic risk factors, excess adiposity, particularly visceral fat, has been linked to direct structural and functional changes within the myocardium, including left ventricular hypertrophy and interstitial fibrosis [7]. These changes lead to increased myocardial stiffness and impaired relaxation, further exacerbated by the metabolically disturbed and pro-inflammatory environment that accompanies obesity [7,10]. Importantly, obesity often coexists with adverse lifestyle behaviors that may further compound cardiovascular risk. Sedentary behavior, excessive alcohol consumption, and tobacco smoking are well-established contributors to cardiovascular disease and can impair endothelial function and cellular metabolism while promoting systemic inflammation [11,12,13,14,15,16]. The convergence of obesity-related and lifestyle-associated stressors may therefore amplify maladaptive cardiac remodeling and accelerate the progression of cardiovascular dysfunction [17,18,19]. Accordingly, addressing obesity together with broader lifestyle-related risk factors is essential for preventing cardiac remodeling and preserving long-term cardiometabolic health.

Beyond its local metabolic effects, obesity is also a well-established driver of gut microbial dysbiosis, characterized by reduced microbial diversity, expansion of pro-inflammatory, endotoxin-producing taxa, and depletion of beneficial, short-chain-fatty-acid (SCFA)-producing bacteria [20,21]. This dysbiotic shift compromises intestinal barrier integrity, permitting translocation of bacterial products such as lipopolysaccharide (LPS) into systemic circulation, a phenomenon known as metabolic endotoxemia [22,23]. Circulating LPS and other dysbiosis-associated metabolites can activate Toll-like receptor 4 (TLR4) and the downstream nuclear factor kappa-light-chain-enhancer of activated B cells (NF-κB) signaling pathway in the myocardium, promoting cardiomyocyte hypertrophy and fibrosis [24,25]. This bidirectional relationship between the gut microbiome and the heart, termed the gut–heart axis, has emerged as a candidate mechanistic link between obesity-associated dysbiosis and pathological cardiac remodeling, raising the possibility that dietary strategies capable of correcting gut dysbiosis could also confer cardiac protection [25,26].

Matcha is a powdered tea produced from shade-grown tea leaves that are rich in chlorophyll and amino acids, giving it a deep green color and a characteristic umami taste [27]. It is widely incorporated into dairy, cereal, bakery, and beverage products, where it enhances sensory quality, antioxidant capacity, nutritional value, and shelf life [28]. Recently, matcha has gained significant attention due to its potential health benefits, including anti-inflammatory, cognitive-enhancing, stress-reducing, anti-obesity, anti-diabetic, and anti-tumorigenic effects [28,29]. These effects are attributed to its bioactive compounds, such as catechins, L-theanine, dietary fiber, and phenolics, which may act as an antioxidant, modulate gut microbiota, and regulate metabolic and inflammatory pathways [28,29]. Notably, catechins and other tea polyphenols have also been shown to exert prebiotic-like and antimicrobial effects within the gut, suppressing pathogenic bacteria while promoting the growth of beneficial taxa such as *Lactobacillus* species [30,31], suggesting that matcha may be capable of correcting the very dysbiotic signature associated with obesity-driven cardiac remodeling.

Despite the wide range of reported bioactivities of matcha and its widespread use as a dietary supplement, no studies have yet investigated its effects on cardiac health under obesogenic conditions. In this study, we examined the effects of matcha supplementation on cardiac remodeling and the gut microbiota in an HFD-fed mouse model to determine whether matcha exerts cardioprotective effects in association with modulation of the gut–heart axis. We hypothesized that matcha supplementation would attenuate HFD-induced cardiac dysfunction and remodeling through both the direct actions of its bioactive compounds on cardiac tissue and the indirect modulation of the gut microbiota.

## 2. Materials and Methods

### 2.1. Animals

All animal experiments were conducted in an Association for Assessment and Accreditation of Laboratory Animal Care (AAALAC)-accredited facility and approved by the Oklahoma State University Institutional Animal Care and Use Committee (IACUC; protocol #21–34). Five-week-old male C57BL/6J mice were obtained from The Jackson Laboratory (Bar Harbor, ME, USA) and acclimated for a week at the Animal Resource Center at Oklahoma State University. After acclimation, baseline body weight and blood glucose levels under fed and 6 h-fasted conditions were measured. The mice (*n* = 29) were then randomly assigned to one of four dietary groups: normal chow diet (NCD; Cat. No. 101845; *n* = 7), high-fat, high-sucrose diet (HFD; Cat. No. 103806; *n* = 7), HFD supplemented with 0.2% matcha (HML; Cat. No. 105343; *n* = 7), or HFD supplemented with 1% matcha (HMH; Cat. No. 105344; *n* = 8). Customized diets, with or without matcha supplementation, were purchased from Dyets Inc. (Bethlehem, PA, USA). Commercially available premium organic ceremonial-grade matcha was purchased from Matcha Organics (Brownwood, TX, USA) and used in the present study. Additional product and sourcing information is available from the manufacturer’s website (https://matchaorganics.com/). The NCD provided 3761.92 kcal/kg, with approximately 61% of total calories derived from carbohydrates and 17% from fat. In comparison, the HFD, HML, and HMH diets provided 4954.58, 4947.67, and 4918.55 kcal/kg, respectively, with approximately 49% of total calories derived from fat and 33–34% from carbohydrates. The HML and HMH diets were prepared using the same HFD formulation, including its sucrose content, with the addition of matcha at the specified concentrations. Detailed compositions of the NCD, HFD, and matcha-supplemented diets are provided in the Supplementary Materials (Tables S1–S4). Mice had ad libitum access to food and water throughout the 10-week experimental period. Food intake and body weight were recorded weekly.

At the end of the 10-week intervention, mice were anesthetized with isoflurane in an induction chamber using a small-animal anesthesia machine (Model R550IE; RWD Life Science Co., Ltd., Shenzhen, China). Adequate anesthesia was confirmed by the absence of a pedal withdrawal reflex. Blood was then collected from the retro-orbital sinus, after which the mice were euthanized by cervical dislocation. Biospecimens, including the heart, liver, and epididymal white adipose tissue (WAT), were collected for further analyses.

### 2.2. Histological Analysis

Heart tissues were paraffin-embedded, sectioned, deparaffinized in xylene, and rehydrated through a graded ethanol series to distilled water. Antigen retrieval was performed in 1× immunohistochemistry Antigen Retrieval Solution, Low pH (Invitrogen, Carlsbad, CA, USA: Cat. No. 00-4955-58) using a boiling water bath for 20 min. After cooling to room temperature, sections were blocked with 10% normal goat serum (Invitrogen, Cat. No. 31873) for 1 h and incubated overnight at 4 °C with Alexa Fluor 488-conjugated wheat germ agglutinin (WGA; 50 μg/mL; Cat, No. W11261). Following incubation, sections were washed three times with 0.2% phosphate-buffered saline containing Tween-20 (PBST) and mounted using a fluorescence-compatible mounting medium. Images were acquired using a Keyence BZ-X710 fluorescence microscope at 20× magnification (KEYENCE Corporation, Osaka, Japan). Cardiomyocyte cross-sectional area was quantified using ImageJ (V1.54p) software (National Institutes of Health, Bethesda, MD, USA) by manually outlining individual WGA-labeled cardiomyocytes with the freehand selection tool. Twenty randomly selected cardiomyocytes were measured per image, and five images were analyzed per mouse, yielding a total of 100 cardiomyocytes per mouse. Only cardiomyocytes exhibiting a transverse orientation and distinct cell boundaries were included. Cardiomyocyte cross-sectional area was expressed in µm^2^. The mean cardiomyocyte cross-sectional area for each animal was calculated and used for subsequent statistical analyses.

For Picrosirius Red staining, paraffin-embedded heart tissues were sectioned at 5 µm thickness. They were then deparaffinized by three consecutive immersions in xylene for 5 min each, followed by rehydration through a graded ethanol series (100% ethanol twice for 3 min each, 95% ethanol for 3 min, 70% ethanol for 3 min) and distilled water (3 min, twice). Sections were then stained using the Mercedes Scientific^®^ Picro-Sirius Red Stain Kit for cardiac muscle (Lakewood Ranch, FL, USA: ref: MER SRC-1) according to the manufacturer’s instructions. Briefly, slides were incubated with 0.2% phosphomolybdic acid solution for 3 min with complete tissue coverage, briefly rinsed in distilled water, and then stained with Picrosirius Red solution for 70 min. Sections were subsequently washed twice in 0.5% acetic acid solution, rinsed in absolute ethanol, dehydrated twice in absolute ethanol, and mounted with Shandon-Mount™ (Thermo Fisher Scientific, Kalamazoo, MI, USA; ref: 1900333). Sections were imaged at 4× magnification using a Keyence BZ-X710 microscope (KEYENCE Corporation, Osaka, Japan), and whole-heart images were generated by automatically stitching individual fields using the built-in software. Cardiac collagen deposition was quantified as previously described by Kammerer et al. 2024 [32], with a FIJI (ImageJ) macro plugin was applied to analyze the entire tissue section without segmentation to assess overall cardiac collagen content, excluding artifacts such as staining debris and tissue folding. The collagen score incorporates both the collagen-positive area and staining saturation, thereby accounting for variation in overall staining intensity, whereas the collagen area fraction represents the ratio of the collagen-positive area to the total tissue area. Additional details regarding the image-analysis macro used to determine these parameters are provided by Kammerer et al. [32].

### 2.3. Microbial Analysis of the Feces

Fresh fecal samples were collected by placing each mouse individually in a sterile paper cup. Fecal pellets were collected aseptically, immediately placed on ice, and stored at −80 °C until genomic DNA (gDNA) extraction. gDNA was extracted using the QIAamp Fast DNA Stool Mini Kit (Qiagen, Hilden, Germany) according to the manufacturer’s instructions. This silica membrane–based procedure includes DNA binding, washing, and elution steps to remove potential contaminants. DNA concentration and purity were assessed using a NanoDrop spectrophotometer, and DNA integrity was further evaluated by Novogene (Sacramento, CA, USA) using an in-house gel-based quality-control procedure. Because all samples submitted for sequencing met the required concentration, purity, and integrity criteria, no additional purification using a separate kit was performed. Samples were required to have a DNA concentration of ≥5 ng/μL and an A260/A280 ratio of 1.8–2.0. Purified gDNA samples were submitted to Novogene Corporation Inc. (Sacramento, CA, USA) for 16S rRNA gene sequencing (*n* = 4–6 per group). Before sequencing, Novogene evaluated DNA integrity using an in-house gel-based quality-control procedure. Only samples that met the concentration, purity, and integrity criteria were processed for sequencing. The sequencing data were deposited in the NCBI Sequence Read Archive under BioProject accession number PRJNA1483701.

For downstream bioinformatic analysis, demultiplexed raw sequencing reads with adapter and primer sequences removed were processed using QIIME2 v2025.4 [33]. Paired-end reads were joined, followed by quality filtering and denoising using the Deblur algorithm to generate high-quality amplicon sequence variants (ASVs) [34]. Taxonomic classification of ASVs was initially performed using the Ribosomal Database Project (RDP) database [35]. The 100 most abundant ASVs were further manually verified and reclassified, when necessary, using the more recent EzBioCloud 16S database (v2025.04.21) [36]. ASVs detected in fewer than 5% of samples were excluded from subsequent analyses.

Microbial diversity analyses and data visualization were conducted in R v4.5.1 using the phyloseq package v1.53.0 [37]. Alpha diversity was assessed using observed ASVs, Shannon diversity index, and Pielou’s evenness index to evaluate microbial richness, diversity, and community evenness. Beta diversity was assessed using weighted and unweighted UniFrac distance metrics to evaluate differences in microbial community composition among experimental groups [38].

### 2.4. Statistical Analysis

All statistical analyses were performed using GraphPad Prism version 9.5.1 (GraphPad Software, Boston, MA, USA). Data distributions were assessed visually using QQ plots. Data were considered approximately normally distributed when the plotted observations closely followed the diagonal reference line without systematic curvature or substantial deviations at the tails. Each animal was considered an independent experimental unit. Potential outliers were identified using GraphPad’s outlier calculator with α = 0.05 and excluded from the analysis. Differences among experimental groups were evaluated using one-way analysis of variance (ANOVA), followed by the appropriate multiple-comparisons post hoc test. Data are presented as the mean ± standard error of the mean (SEM), and statistical significance was defined as *p* < 0.05.

Bioinformatic data analysis and visualization were implemented in R v4.5.1 with the RStudio integrated development environment (IDE) v2025.05.0 (RStudio, Boston, MA, USA). Statistical significance was measured using parametric or non-parametric methods, depending on the normality of the data determined by the Shapiro–Wilk test and homogeneity of variance across groups determined by Levene’s test. One-way ANOVA and post hoc Tukey’s test were used for the parametric methods, while the Kruskal–Wallis test and post hoc Dunn’s test were used for the non-parametric methods. Homogeneity of multivariate dispersion was assessed using the betadisper function implemented in the vegan package, followed by a permutation test with 999 permutations. Bacterial β-diversity was then compared among groups using permutational multivariate analysis of variance (PERMANOVA) with 999 permutations, also implemented in the vegan package (v2.7-1) in R. ANCOM-BC2 (v2.12.0) was used for differential abundance analysis with the following parameters: prv_cut = 0, *p*_adj_method = “fdr”, pseudo = 1, pseudo_sens = FALSE, and pairwise = TRUE; all other parameters were left at their default settings. *p*-values were adjusted for multiple comparisons using the false discovery rate (FDR) method where applicable. An adjusted *p*-value < 0.05 was considered statistically significant.

## 3. Results

### 3.1. Matcha Supplementation Reduced Heart Weight in HFD-Fed Mice

**Figure 1:**
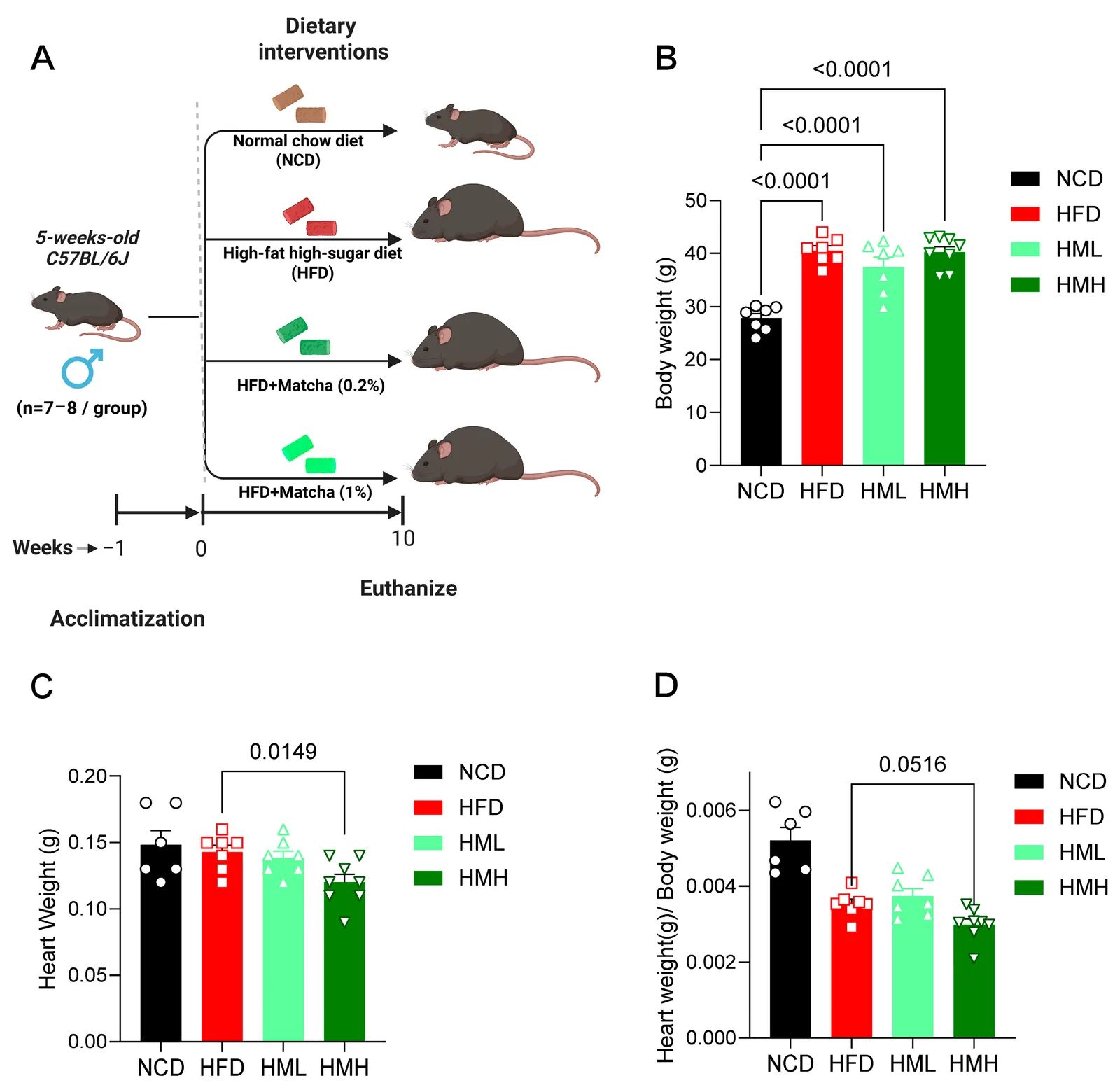
Matcha supplementation attenuated heart weight in HFD-fed obese mice. (**A**) Experimental design. Six-week-old male C57BL/6J mice were fed either an NCD or an HFD for 10 weeks. HFD-fed mice received either no supplementation (HFD), 0.2% matcha (HML), or 1% matcha (HMH). (**B**) Final body weight after 10 weeks of dietary intervention. (**C**) Heart weight. (**D**) Heart weight normalized to body weight. Data are presented as the mean ± SEM (*n* = 6–8 mice per group). Statistical significance was determined by one-way ANOVA followed by a multiple-comparison test. *p* < 0.05 was considered statistically significant. (NCD: normal chow diet; HFD: high-fat, high-sugar diet; HML: high-fat, high-sugar diet with 0.2% matcha; HMH: high-fat, high-sugar diet with 1% matcha). Illustration (**A**) was created in BioRender.com. Lamichhane, G. (2026) https://BioRender.com/fsheydg (accessed on 6 September 2026).

After 10 weeks of dietary intervention, all HFD-fed groups, including those receiving matcha supplementation, exhibited significantly higher body weight than the NCD control group, confirming successful induction of obesity by the HFD (Figure 1B) (F = 23.38; ANOVA *p* < 0.0001; *df* = 28). Further details about the metabolic phenotype of mice after a 10-week intervention can be found in Supplementary Figures S1 and S2. Neither 0.2% nor 1% matcha supplementation attenuated HFD-induced body weight gain. Despite having no effect on body weight, mice receiving 1% matcha supplementation showed significantly lower heart weight among the high-fat diet-fed cohort compared with the HFD control group (Figure 1C; *p* = 0.0149) (F = 5.052; ANOVA *p* = 0.0174; *df* = 21). Similarly, the heart weight-to-body weight ratio showed a trend towards significant reduction (*p* = 0.0516) in the 1% matcha group relative to HFD controls (Figure 1D) (F = 5.863; ANOVA *p* = 0.0104; *df* = 21). In contrast, supplementation with 0.2% matcha did not significantly affect either heart weight or the heart weight-to-body weight ratio. This prompted us to further investigate the effects of matcha supplementation on cardiomyocyte morphology.

### 3.2. Matcha Supplementation Attenuated HFD-Induced Cardiomyocyte Hypertrophy in Mice

**Figure 2:**
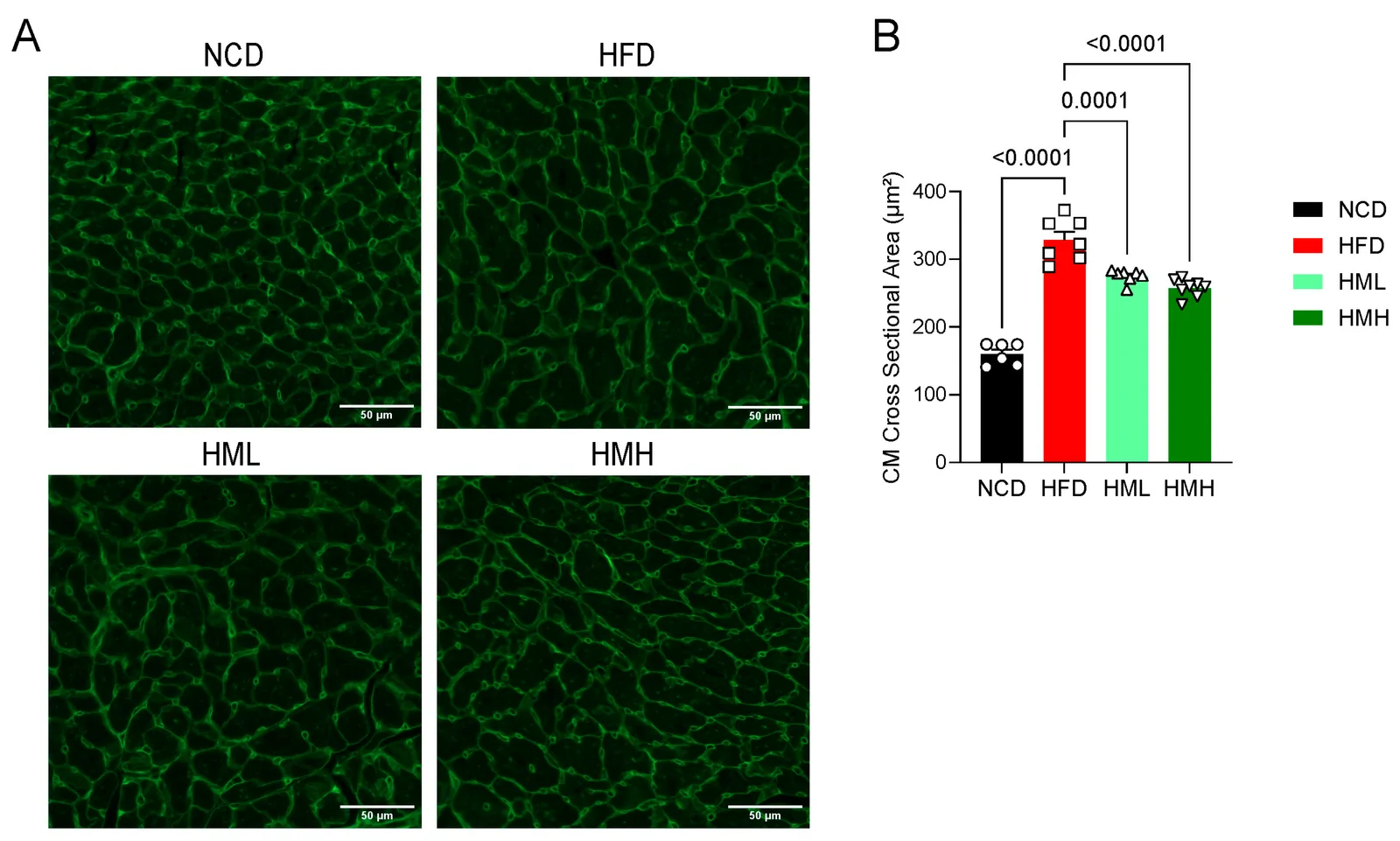
Matcha supplementation reduced HFD-induced cardiac hypertrophy in mice. (**A**) Representative WGA-stained cardiac sections showing cardiomyocyte morphology. (**B**) Quantification of cardiomyocyte cross-sectional area. Data are presented as the mean ± SEM (*n* = 6–8 mice per group). Statistical significance was determined by one-way ANOVA followed by a multiple-comparison test. *p* < 0.05 was considered statistically significant. (NCD: normal chow diet; HFD: high-fat, high-sugar diet; HML: high-fat, high-sugar diet with 0.2% matcha; HMH: high-fat, high-sugar diet with 1% matcha; WGA: wheat germ agglutinin).

Histological examination of heart sections stained with WGA revealed a marked increase in cardiomyocyte size in HFD-fed mice compared with the NCD group, indicating the development of cardiomyocyte hypertrophy (Figure 2A). In contrast, cardiomyocyte size appeared small in both the 0.2% and 1% matcha-supplemented groups. Quantitative analysis of cardiomyocyte cross-sectional area confirmed these observations, with the HFD control group exhibiting the largest cardiomyocyte cross-sectional area (Figure 2B). Both matcha-supplemented groups showed significantly reduced cardiomyocyte cross-sectional area compared with HFD controls, with the 1% matcha group demonstrating a greater reduction than the 0.2% group (F = 86.12; ANOVA *p* < 0.0001; *df* = 27). These findings suggest that dietary matcha supplementation attenuates HFD-induced cardiomyocyte hypertrophy. This result was further supported by a significant decrease in LV wall thickness in HML- and HMH-supplemented mice (Supplementary Figure S3).

### 3.3. Matcha Supplementation Ameliorated HFD-Induced Cardiac Fibrosis in Mice

**Figure 3:**
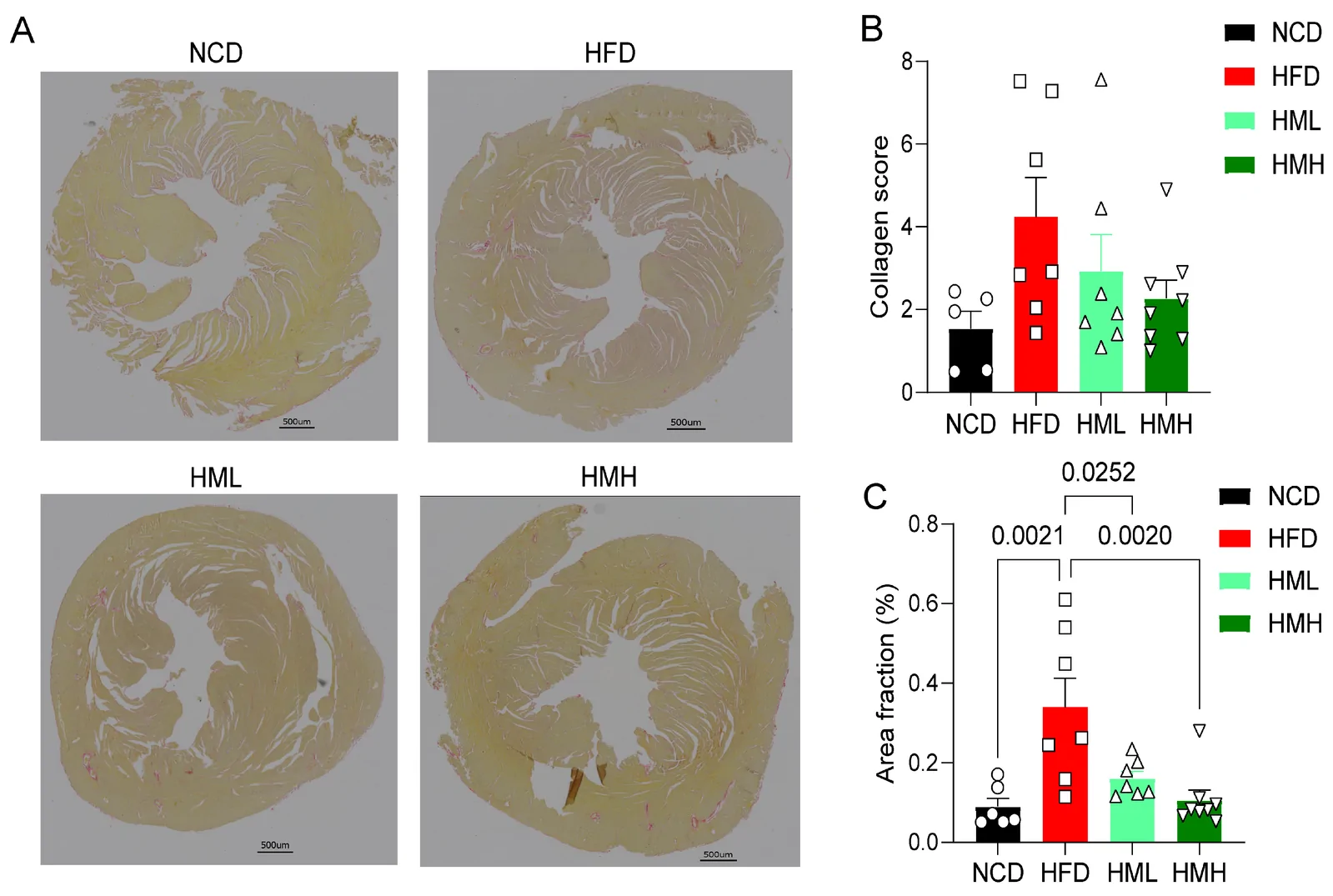
Matcha supplementation attenuated HFD-induced cardiac fibrosis in mice. (**A**) Representative Picrosirius Red-stained heart sections showing cardiac collagen deposition. Quantification of the collagen score (**B**) and collagen-positive area fraction (**C**). Data are presented as the mean ± SEM (*n* = 5–8 mice per group). Statistical significance was determined by one-way ANOVA followed by a multiple-comparison test. (NCD: normal chow diet; HFD: high-fat, high-sugar diet; HML: high-fat, high-sugar diet with 0.2% matcha; HMH: high-fat, high-sugar diet with 1% matcha).

As long-term cardiac hypertrophy can lead to cardiac fibrosis [39], we next performed Picrosirius Red staining of heart sections to evaluate collagen deposition. Our results revealed a significant increase in collagen deposition in HFD-fed mice compared with the NCD group, indicating increased cardiac fibrosis under metabolic stress (Figure 3A). Quantitative analysis of the collagen-positive area fraction demonstrated that both the 0.2% and 1% matcha-supplemented groups had significantly lower cardiac collagen deposition than the HFD control group (Figure 3C; F = 7.651; ANOVA *p* = 0.0009; *df* = 27). The reduction in collagen accumulation indicates that matcha plays a role in ameliorating HFD-induced cardiac fibrosis.

### 3.4. Matcha Prevented HFD-Induced Imbalance of Microbiome in Mice

**Figure 4:**
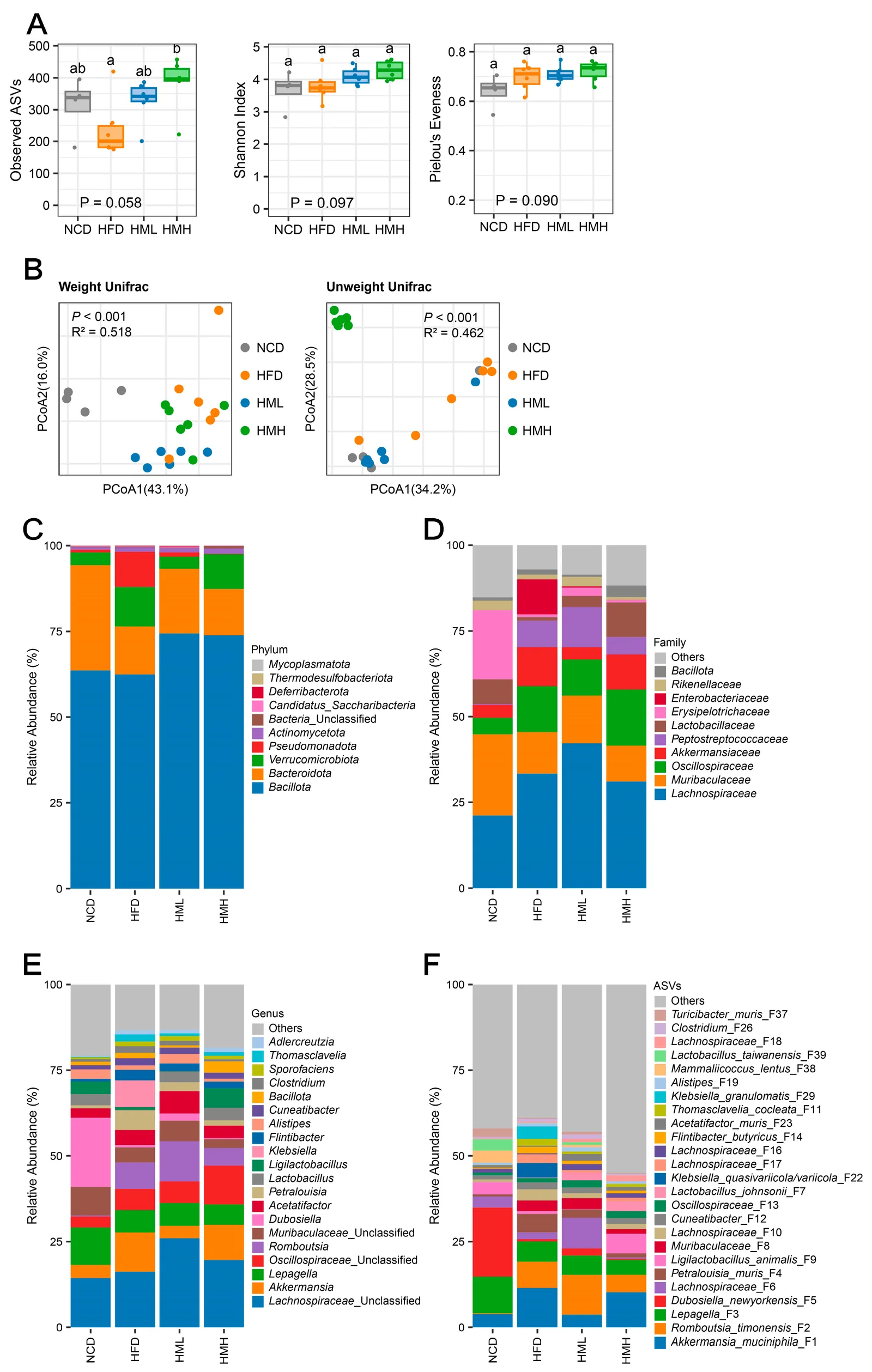
Matcha supplementation altered the gut microbiome composition in mice. (**A**) Alpha diversity of the fecal gut microbiota, represented by the observed ASV’s, Shannon index, and Pielou’s evenness index. (**B**) Beta diversity, represented by weighted and unweighted UniFrac distances. Relative taxonomic abundance of the fecal gut microbiota at the (**C**) phylum, (**D**) family, (**E**) genus, and (**F**) ASV levels (*n* = 4–6 mice per group). (NCD: normal chow diet; HFD: high-fat, high-sugar diet; HML: high-fat, high-sugar diet with 0.2% matcha; HMH: high-fat, high-sugar diet with 1% matcha). In (**A**), group with different lowercase letters (a, b) are significantly different from each other (*p* < 0.05), whereas group sharing the same letter are not significantly different.

After 10 weeks of HFD intervention, alpha diversity analysis of the fecal gut microbiota revealed a marked reduction in microbial richness, as measured by observed ASVs, in HFD-fed mice compared with the NCD group (Figure 4A). In contrast, the number of observed ASVs in both the low-dose matcha (HML) and high-dose matcha (HMH) groups was comparable to those in the NCD group, suggesting that dietary matcha supplementation prevented the HFD-induced loss of microbial richness. However, the Shannon diversity index and Pielou’s evenness index remained unchanged among the experimental groups, indicating that dietary interventions had minimal effects on microbial diversity and evenness.

Beta diversity analysis based on weighted and unweighted UniFrac distances demonstrated distinct clustering of the NCD, HFD, HML, and HMH groups in the PCoA plot (Figure 4B). The NCD, HML, and HMH groups were mostly distributed towards the left side of the PCoA plot, whereas the HFD group clustered on the right side. Pairwise distance analysis showed the greatest separation between HMH and HFD groups, as well as between NCD and HFD groups, while the NCD and HML groups exhibited the smallest distance, consistent with their overlapping distribution in the PCoA plot. Details of PERMANOVA results for pairwise comparison of microbiota in weighted and unweighted UniFrac analysis can be found in Supplementary Table S5.

Analysis of the relative abundance of the fecal gut microbiota across treatment groups showed distinct taxonomic profiles at the phylum, family, genus, and ASV levels (Figure 4C–F). At the phylum level, the relative abundance of *Pseudomonadota* was markedly increased in HFD-fed mice compared with the NCD group. This increase was attenuated in both matcha-supplemented groups (Figure 4C).

At the family level, the HFD-fed mice exhibited a higher relative abundance of *Enterobacteriaceae* than the NCD-fed mice (Figure 4D). In contrast, both matcha-treated groups showed a reduced abundance of *Enterobacteriaceae*. Conversely, members of the *Lactobacillaceae* family displayed the opposite trend. The relative abundance of *Lactobacillaceae* was substantially lower in the HFD group than in the NCD group, whereas supplementation with matcha restored its abundance in both the HML and HMH groups.

A similar pattern was observed at the genus level. The relative abundances of the beneficial genera, including *Lactobacillus* and *Ligilactobacillus*, were markedly elevated in the HMH group but remained low in the HFD group (Figure 4E). In contrast, the relative abundance of the potentially pathogenic genus *Klebsiella* was increased in HFD-fed mice and reduced by matcha supplementation at both doses.

At the ASV level, beneficial species, including *Lactobacillus johnsonii* and *Lactobacillus animalis*, were enriched in the matcha-treated groups, whereas the potentially pathogenic species *Klebsiella quasivariicola* was more abundant in the HFD group and decreased following matcha supplementation (Figure 4F). Collectively, these findings suggest that dietary matcha supplementation partially mitigated HFD-induced gut microbiota dysbiosis by reducing the relative abundance of potentially pathogenic bacteria while promoting the enrichment of beneficial microbial taxa.

**Figure 5:**
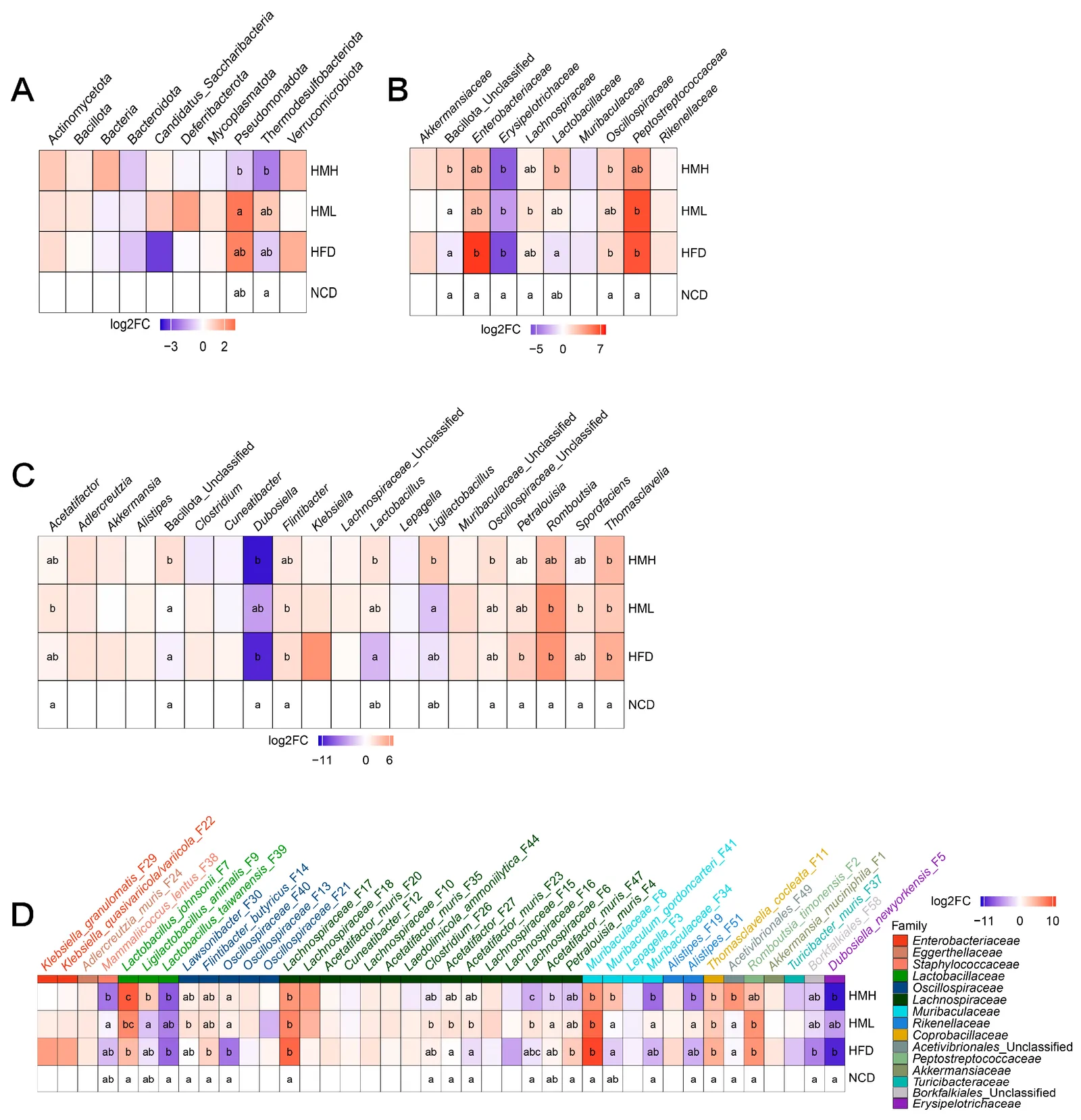
Matcha supplementation altered the gut microbiota composition in HFD-fed mice. Differentially abundant taxa identified by ANCOM-BC2 at the (**A**) phylum, (**B**) family, (**C**) genus, and (**D**) ASV levels. Groups labeled with different letters (a, b, and c) are significantly different from one another (*p* < 0.05; *n* = 4–6 mice per group). (NCD: normal chow diet; HFD: high-fat, high-sugar diet; HML: high-fat, high-sugar diet with 0.2% matcha; HMH: high-fat, high-sugar diet with 1% matcha).

Differential abundance analysis using ANCOM-BC2 revealed no significant differences among the treatment groups at the phylum level for the 10 most abundant phyla (Figure 5A). However, significant alterations were observed at lower taxonomic levels. At the family level, the relative abundances of *Bacillota* unclassified taxa and *Lactobacillaceae* were significantly higher in the HMH group than in the HFD group (Figure 5B). Similarly, at the genus level, unclassified members of *Bacillota* and the genus *Lactobacillus* were significantly enriched in the HMH group relative to the HFD group (Figure 5C). These findings suggest that high-dose matcha supplementation selectively promoted beneficial bacterial populations that were depleted by HFD feeding. Further ANCOM-BC2 analysis of the 40 most abundant ASVs identified several taxa that differed significantly among the treatment groups (Figure 5D). Notably, the HMH group exhibited a significantly higher abundance of *Lactobacillus johnsonii* compared with the HFD group. In addition, ASVs belonging to *Oscillospiraceae* (F40) were significantly enriched in both matcha-treated groups (HML and HMH) relative to the HFD group. The abundances of *Muribaculum gordoncarteri* (F41) and *Actinivibrionales* (F49) were also significantly increased in the HMH group compared with the HFD group.

Collectively, these results indicate that matcha supplementation, particularly at the higher dose, selectively promoted the enrichment of beneficial microbial taxa associated with gut health and may have contributed to the restoration of gut microbial homeostasis disrupted by HFD feeding.

**Figure 6:**
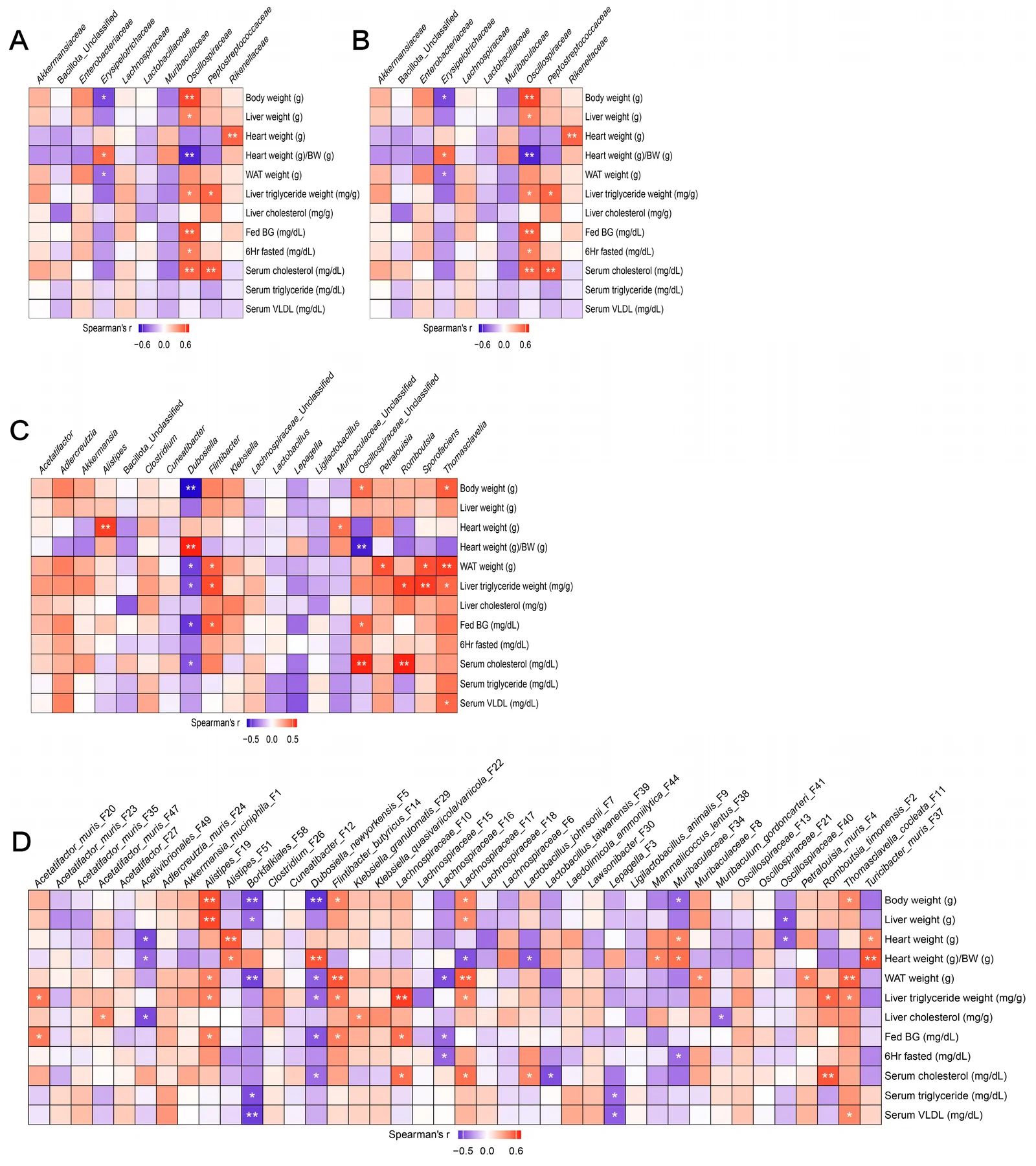
Spearman correlation analysis between metabolic and cardiac health parameters and the fecal gut microbiota. Correlation analysis at the (**A**) phylum, (**B**) family, (**C**) genus, and (**D**) ASV levels. All treatment groups were pooled for the correlation analysis. To account for multiple correlation testing, the resulting *p*-values were adjusted using the FDR method. Asterisks indicate statistically significant correlations (* *p* < 0.05; ** *p* < 0.01; *n* = 4–6 mice per group).

Interestingly, correlation analysis demonstrated that *Lactobacillus johnsonii* and *Actinivibrionales* (F49), both significantly enriched in the HMH group, were negatively correlated with the heart weight-to-body weight ratio (Figure 6D). These results indicate that higher relative abundances of these taxa were associated with lower heart weight.

## 4. Discussion

Obesity is a well-established driver of pathological cardiac remodeling, and lifestyle interventions, including diet and exercise, remain important strategies for preventing its progression [40,41]. In the present study, we demonstrate that dietary matcha supplementation attenuated several hallmarks of obesity-associated cardiac remodeling in HFD-fed mice. Specifically, matcha supplementation reduced heart weight, attenuated cardiomyocyte hypertrophy, and decreased cardiac collagen deposition without affecting HFD-induced body weight gain. In parallel, matcha supplementation reshaped the fecal microbiota by restoring microbial richness, suppressing potentially pathogenic, endotoxin-associated taxa such as *Klebsiella* and *Enterobacteriaceae* [42,43], and enriching beneficial taxa including *Lactobacillus johnsonii* [44]. Notably, the relative abundance of *L. johnsonii* and an ASV within *Actinivibrionales* (F49) were negatively correlated with heart weight, providing correlative evidence linking alterations in the gut microbiota with the observed cardioprotective phenotype. These findings demonstrate that matcha supplementation was accompanied by both attenuation of cardiac remodeling and alterations in fecal microbial composition in HFD-fed mice. However, the associations between microbial taxa and cardiac phenotypes are correlative and do not establish a causal or mediating role for the gut microbiota. Further mechanistic studies, such as microbiota-transfer or depletion experiments, are needed to determine whether the observed microbial alterations contribute to the cardiac effects of matcha.

Previous studies have reported that matcha supplementation reduces body weight in obese animal models [45,46]. In our study, matcha supplementation did not significantly affect body weight gain in mice despite improving cardiac structure. This discrepancy may be attributable to differences in the type of matcha used, the supplementation dose, the dietary composition, or the duration of the intervention. A notable feature of our results is that matcha supplementation improved cardiac remodeling independently of changes in body weight. This dissociation suggests that the cardioprotective effects of matcha are not simply secondary to reduced adiposity but may involve direct actions on the myocardium or modulation of pathways linking metabolic stress to cardiac remodeling. This interpretation is consistent with previous studies showing that green tea catechins, particularly epigallocatechin-3-gallate (EGCG), act directly on cardiomyocytes and vascular tissues to protect against atherosclerosis, endothelial dysfunction, and cardiomyocyte hypertrophy [47,48,49]. Wan et al. also demonstrated that EGCG attenuated high-glucose-induced cardiomyocyte hypertrophy, inflammation, and oxidative stress [50]. In addition, improvements in cardiometabolic risk factors induced by green tea may contribute to the cardioprotective effects of matcha [51,52]. Changes in gut microbial composition and associated gut-derived factors may also influence systemic inflammatory and metabolic signaling. However, these potential mechanisms were not directly evaluated in the present study and require further investigation. Our histological findings extend these direct mechanisms of bioactive compounds to a dietary food matrix (matcha) in an obesogenic environment. We demonstrated this effect using histology of the heart to observe cardiomyocyte structure and fibrosis. We observed a significant increase in cardiomyocyte hypertrophy and fibrosis after 10 weeks of HFD supplementation. This observation is consistent with previous findings where several studies supplementing a cholesterol-enriched diet or high-fat diet for a minimum of 5 to up to 20 weeks showed significant cardiomyocyte hypertrophy and fibrosis [53,54,55]. Interestingly, matcha supplementation ameliorated cardiac hypertrophy, LV wall thickness, and fibrosis significantly at both doses. No significant reduction in heart weight or the heart weight-to-body weight ratio in the 0.2% matcha-supplemented group suggests that a reduction in heart weight *per se* is not necessary for matcha to produce beneficial microstructural changes within the heart.

High-fat, high-sugar diet-induced obesity has been consistently shown to alter the gut microbiome in mice, known as dysbiosis [56,57,58]. For example, Do et al. showed reduced microbial diversity and distinctive microbial composition, as represented by reduced operational taxonomic units (OTUs) and the Shannon diversity index, together with distinct clustering of microbial communities in a PCoA plot in dietarily challenged mouse [56]. Similarly, Kong et al. also demonstrated reduced microbial diversity in mice fed a high-fat, high-sucrose diet [57]. Our 16S rRNA sequencing data also mirrored this pattern, as the HFD group showed reduced numbers of observed ASVs and distinct clustering in the PCoA plot compared with the NCD group. This effect on the microbiome was partially reversed by matcha supplementation, as indicated by increased ASVs and distinctive distribution of treatment groups in the PCoA plot.

Relative abundance profiles at the phylum, family, genus, and species levels revealed several noteworthy patterns. At the phylum level, *Pseudomonadota* (formally known as *Proteobacteria*), a known group of opportunistic pathobionts and endotoxin producers, was markedly increased in the HFD group compared to the NCD group, while matcha treatment at both doses showed lower abundance of that phylum [59]. Proteobacteria has been previously shown as an independent marker of cardiovascular risk [60]. At the family level, we consistently observed higher relative abundance of the *Enterobacteriaceae* family, a member of the *Pseudomonadota* phylum, under HFD feeding. This family harbors pathobionts that are implicated in inflammatory bowel diseases [61]. *Enterobacteriaceae* were also found to be increased in patients with atherosclerotic CVDs [62]. Its abundance was reduced in both matcha-supplemented groups. A similar pattern was observed at the genus level: *Klebsiella*, a potentially pathogenic member of the *Enterobacteriaceae* family, was enriched in HFD-fed mice but reduced following matcha supplementation [63,64]. *Klebsiella* has also been associated with hypertension [65]. Therefore, the reduction in these potentially pathogenic taxa may decrease gut-derived endotoxin exposure and systemic inflammatory signaling, which could otherwise contribute to cardiomyocyte hypertrophy and cardiac remodeling. However, circulating endotoxin levels, intestinal barrier integrity, and inflammatory signaling were not directly assessed; therefore, this proposed mechanism remains to be established experimentally. On the other hand, the HFD group displayed a lower abundance of the *Lactobacillaceae* family, a well-known bacterial family known for probiotic effects that promote gut health and help maintain intestinal homeostasis [66]. Supplementation with matcha at both doses restored this imbalance by increasing their abundance. This alteration in *Lactobacillaceae* also translated downstream at the genus level, where abundance of *Lactobacillus* and *Ligilactobacillus* was lower in the HFD group compared to the NCD group, whereas HML successfully restored *Lactobacillus* while HMH restored both genera. A similar pattern of changes was also observed at the ASV level for *Lactobacillus johnsonii*, *Lactobacillus animalis*, and *Klebsiella quasivariicola.*

Furthermore, ANCOM-BC2 analysis clearly showed a specific pattern for the HMH group compared to the HFD group. HMH showed lower *Thermodesulfobacteriota* (vs NCD) and *Pseudomonadota* (vs HML). The former (*Thermodesulfobacteriota* at phylum) is known to contain bacteria that metabolize sulfur and contribute to intestinal inflammation and barrier damage [67]. The difference was found to be more obvious at lower taxonomic levels, as we observed significantly higher abundances of *Bacillota*_unclassified and *Lactobacillaceae* in the HMH group compared with the HFD group. At the genus level, *Bacillota*_unclassified and *Lactobacillus* were significantly enriched in the HMH group relative to the HFD group. ANCOM-BC2 analysis of the top 40 ASVs also showed that HMH had a strong influence on the microbiota. In the HMH group, *Lactobacillus johnsonii*, *Oscillospiraceae* (F40), *Muribaculum gordoncarteri* (F41), and *Actinivibrionales* (F49) were significantly increased. HML, however, showed increased abundance of *Oscillospiraceae* (F40), *Acetatitifactor*_F27, and *Acetatitifactor_muris*_F23. The 1% matcha group exhibited a greater number of alterations in gut microbial composition than the lower-dose group, suggesting a broader compositional response to the higher dose. However, a greater number of altered taxa does not necessarily indicate a stronger beneficial or functional effect. Regarding cardiac outcomes, 1% matcha significantly reduced heart weight compared with the HFD group, whereas the lower dose did not. In contrast, both matcha doses reduced cardiomyocyte size. Thus, the microbiota and cardiac responses were not fully concordant across doses, and further functional and mechanistic analyses are required to determine the biological significance of the dose-dependent microbial changes. The negative correlation between heart weight/body weight and *Lactobacillus johnsonii*, one of the ASVs enriched by 1% matcha, aligns with prior evidence of this species’ cardioprotective role. In a rat myocardial infarction model, gut enrichment of *L. johnsonii* preserved cardiac function and reduced injury cytokines, an effect that was lost after depletion of this microbiota with antibiotics [44]. As our study is correlative (not causal), future studies should evaluate the causal relation of *L. johnsonii* to cardioprotection under dietary-challenged conditions. We also observed increased *Actinivibrionales* (F49) in the HMH group relative to all other groups, and this taxon showed a significant negative correlation with both heart weight and heart weight/body weight, making it a candidate worth further investigation.

Several limitations should be considered when interpreting our findings. First, the association between the gut microbiota and cardiac phenotypes observed in this study is correlative. As we did not perform mechanistic interventions, such as fecal microbiota transplantation, antibiotic-mediated microbiota depletion, or monoassociation with the specific taxa identified here, such experiments would be required to establish causality in our model. Second, we did not directly measure circulating microbial metabolites (e.g., SCFAs, LPS, or trimethylamine *N*-oxide (TMAO)) or markers of intestinal barrier integrity in this study. Quantification of these mediators would provide substantial mechanistic insight into the proposed gut–heart axis linking HFD-induced gut microbiota dysbiosis to cardiac remodeling. Third, this study was conducted exclusively in male mice over a single 10-week intervention period. Therefore, whether these findings are generalizable to female mice, different durations or severities of obesogenic challenges, or human populations remains to be determined. Fourth, we used a commercially available matcha product and did not independently determine its batch-specific concentrations of catechins, EGCG, L-theanine, caffeine, or total polyphenols. In addition, substantial digging and food spillage by the HFHSD-fed mice, including those receiving matcha, prevented reliable quantification of food consumption. Therefore, actual matcha intake in mg/kg body weight/day could not be accurately calculated, and the doses can only be reported as their nominal dietary concentrations. Finally, cardiac assessment in the present study was limited to structural and histomorphometric outcomes, including heart weight, cardiomyocyte size, collagen deposition, LV wall thickness, and LV chamber area. Functional measurements, such as echocardiographic or hemodynamic assessments, were not performed. Therefore, whether the attenuation of cardiac structural remodeling following matcha supplementation translates into improved cardiac function remains to be determined.

## 5. Conclusions

In summary, dietary matcha supplementation attenuated cardiac hypertrophy and fibrosis in a diet-induced obesity model and was associated with alterations in gut microbiota composition. However, the present findings do not establish that these microbial changes mediate the observed cardiac effects. The gut–heart axis represents a potential mechanism through which matcha may influence obesity-associated cardiac remodeling. Further studies incorporating both male and female mice, microbial metabolite profiling, assessment of intestinal barrier function, and causal manipulation of the implicated microbial taxa are needed to determine the mechanistic basis of these associations and evaluate their translational relevance.

## Data Availability

The raw sequencing reads for 16S rRNA sequencing were deposited in the NCBI Sequence Read Archive database under the same BioProject accession number PRJNA1483701. All the datasets used and/or analyzed during the current study are available from the corresponding authors upon reasonable request.

## Supplementary Materials

The following supporting information can be downloaded at: https://www.mdpi.com/article/10.3390/nu18182961/s1, Supplementary methods provide details of (a) serum cholesterol analysis, (b) hepatic cholesterol and triglyceride analysis [68], and (c) H and E staining of heart in details; Table S1: Diet composition of normal chow diet (NCD); Table S2: Diet composition of high-fat high-sugar diet (HFD); Table S3: Diet composition of HFD with 0.2% matcha (HML); Table S4: Diet composition of HFD with 1% matcha (HMH); Figure S1: Body weight (A), body weight gain (B), liver weight (C), WAT weight (D), fed blood glucose level (E), 6hr fasted blood glucose level (F), serum cholesterol level (G) serum triglyceride levels (H) and serum VLDL level (I) of mice under 10-week matcha supplementation; Figure S2: Hepatic triglyceride (A), and hepatic cholesterol (B) of mice under 10-week matcha supplementation. Figure S3: H and E staining images of heart under different treatment condition (A), LV wall thickness in mm for different treatment group (B) and LV chamber area in mm^2^ for heart under different treatment condition (C); Table S5: Analysis of multivariate dispersion of microbiota during beta diversity analysis; Table S6: Pairwise PERMANOVA comparisons of gut microbiota community based on weighted and unweighted UniFrac distance matrices.

## Abbreviations

The following abbreviations are used in this manuscript:

AAALAC	Association for Assessment and Accreditation of Laboratory Animal Care International
ANCOM-BC2	Analysis of Composition of Microbiomes with Bias Correction 2
ANOVA	Analysis of Variance
ASVs	Amplicon Sequence Variants
C57BL/6J	C57 Black 6J (inbred laboratory mouse strain)
CVDs	Cardiovascular Diseases
EGCG	Epigallocatechin-3-gallate
gDNA	Genomic DNA
HFD	High-Fat, High-Sugar Diet
HMH	High-Fat, High-Sugar Diet with 1% matcha
HML	High-Fat, High-Sugar Diet with 0.2% matcha
IACUC	Institutional Animal Care and Use Committee
IHC	Immunohistochemistry
LPS	Lipopolysaccharide
NCBI	National Center for Biotechnology Information
NCD	Normal Chow Diet
NF-κB	Nuclear Factor Kappa-Light-Chain-Enhancer of Activated B Cells
OUT	Operational Taxonomic Unit
PBST	Phosphate-Buffered Saline with Tween 20
PCoA	Principal Coordinates Analysis
SCFA	Short-Chain Fatty Acid
SEM	Standard Error of the Mean
SRA	Sequence Read Archive
TLR4	Toll-Like Receptor 4
TMAO	Trimethylamine *N*-oxide
WGA	Wheat Germ Agglutinin
