# Supplementary material for: Dietary Matcha Attenuates High-Fat High-Sugar Diet-Induced Cardiomyocyte Hypertrophy in Association with Gut Microbiota Remodeling in Mice
Source: Nutrients. 2026 Sep 9;18(18):2961. doi: 10.3390/nu18182961 (PMC13610588; doi:10.3390/nu18182961)
Supplement: Supplementary file 1 [file nutrients-18-02961-s001.zip › nutrients-4505568-supplementary.pdf]

nutrients

MDPI

Supplementary materials

Dietary Matcha Attenuates High-Fat High-Sugar Diet–Induced Cardiomyocyte Hypertrophy by Reshaping the Gut Microbiota in Mice

Gopal Lamichhane [1], Jong-Beom Jin [1], Jiaqing Guo [2], Da-Yeon Lee [1], Femi Olawale [1], Cody Moss [1], Guolong Zhang [2], Jiyoung Bae [1], Edralin A. Lucas [1] and Yoo Kim [1,*]

[1] Department of Nutritional Sciences, Oklahoma State University, Stillwater, OK 74078, USA; gopal.lamichhane@okstate.edu (G.L.); jojin@okstate.edu (J.-B.J.); dayeon.lee@okstate.edu (D.-Y.L.); femi.olawale@okstate.edu (F.O.); cody.moss10@okstate.edu (C.M.); jiyoung.bae@okstate.edu (J.B.); edralin.a.lucas@okstate.edu (E.A.L.)
[2] Department of Animal and Food Sciences, Oklahoma State University, Stillwater, OK 74078, USA; jiaqing.guo@okstate.edu (J.G.); glenn.zhang@okstate.edu (G.Z.)
* Correspondence: yoo.kim@okstate.edu

1. Supplementary material and methods

1.1 Serum cholesterol analysis

Serum cholesterol level were measured using Piccolo Xpress Clinical Chemistry analyzer (Abbott, Chicago, IL, USA) using Lipid Panel reagent discs (Abaxix inc, Union City, CA, USA: ref 400-0025).

1.2 Hepatic cholesterol and triglyceride analysis

Hepatic triglyceride and cholesterol were determined following methods as described previously [1]

1.3 H and E staining

For H and E staining, formalin-fixed, paraffin-embedded tissue sections (4 µm) were deparaffinized in xylene (3 × 3 min) and rehydrated through a graded ethanol series (100% and 95%, 2 changes each, 10 dips per change) followed by distilled water. Sections were stained with Mayer Modified Hematoxylin (Newcomer Supply; Waunakee, WI, USA; Part #1202) for 15 min, washed in tap water (3 min), and blued under running tap water 10 min until nuclei changed from purple to blue. Slides were rinsed in distilled water, dehydrated briefly in 70% ethanol (10 dips), and counterstained with Eosin working solution for 30 sec. Sections were then dehydrated through 95% ethanol (2 × 1 min) and 100% ethanol (2 × 10 dips), cleared in xylene (3 × 10 dips), and coverslipped using a mounting medium. Whole-heart H&E-stained sections were imaged using Keyence BZ-X710 microscope, scale calibrated and analyzed using ImageJ/Fiji. Left ventricular (LV) wall thickness was measured using the straight-line tool, drawn from the endocardial to epicardial border, with multiple measurements taken from both the upper and lower regions of the LV wall and averaged per section. LV chamber area was determined by manually tracing the endocardial boundary using the freehand selection tool, followed by area quantification (Analyze → Measure). For each mouse, two sections (upper and lower regions) were analyzed, and values were averaged to yield a single measurement per animal. LV wall thickness and chamber area are reported in mm and mm^2, respectively.

1.4 Diet composition

Detail dietary composition is provided in tables listed below:

 https://doi.org/10.3390/nu18182961

Table S1: Diet composition of normal chow diet (NCD)

DYET# 101845

Control Diet for Fat/Glucose/Ketosis Study

Ingredient	kcal./gm	grams/kg	kcal./kg
High Nitrogen Casein	3.58	200	716
L-Cystine	4	3	12
Sucrose	4	90	360
Cornstarch	3.6	398.562	1434.8232
Dyetrose	3.8	132	501.6
Soybean Oil	9	70	630
t-Butylhydroquinone	0	0.014	0
Ethoxyquin	0	0.024	0
Cellulose	0	50	0
Mineral Mix #210025	0.88	35	30.8
Vitamin Mix # 310025	3.87	10	38.7
Supplement #410750	3.8	10	38
Choline Chloride	0	1.4	0
		**1000.00**	**3761.9232**

https://doi.org/10.3390/nu18182961

Table S2 Diet composition of high-fat high-sugar diet (HFD)

DYET# 103806

Custom High Fat/High Glucose Diet
(49% Fat Derived Calories, 22% Glucose Derived Calories)

Ingredient	kcal./gm	grams/kg	kcal./kg
High Nitrogen Casein	3.58	200	716
L-Cystine	4	3	12
Sucrose	4	90	360
Cornstarch	3.6	0	0.000
Dyetrose	3.8	50	190
Dextrose	3.64	310.462	1130.1
Soybean Oil	9	70	630
t-Butylhydroquinone	0	0.014	0
Whole Butter (18% Water)	9	181	1629
Ethoxyquin	0	0.024	0
Lard	9	20	180
Cellulose	0	50	0
Mineral Mix #210025	0.88	35	30.8
Vitamin Mix # 310025	3.87	10	38.7
Supplement #410750	3.8	10	38
Choline Chloride	0	1.4	0
Red Dye	0	0.1	0
(roughly 31 grams of water will evaporate)		**1031.000**	**4954.582**

Table S3: Diet composition of HFD with 0.2% matcha (HML)

DYET# 105343

Custom High Fat/High Glucose Diet (#103806)
with 0.2% Matcha

Ingredient	kcal./gm	grams/kg	kcal./kg
High Nitrogen Casein	3.58	200	716
L-Cystine	4	3	12
Sucrose	4	90	360
Cornstarch	3.6	0	0.000
Dyetrose	3.8	50	190
Dextrose	3.64	308.562	1123.2
Soybean Oil	9	70	630
t-Butylhydroquinone	0	0.014	0
Whole Butter (18% Water)	9	181	1629
Ethoxyquin	0	0.024	0
Lard	9	20	180
Cellulose	0	50	0
Mineral Mix #210025	0.88	35	30.8
Vitamin Mix #310025	3.87	10	38.7
Supplement #410750	3.8	10	38
Choline Chloride	0	1.4	0
Matcha (provided)	0	2	0
(roughly 31 grams of water will evaporate)		**1031.000**	**4947.666**

https://doi.org/10.3390/nu18182961

Table S4: Diet composition of HFD with 1% matcha (HMH)

DYET# 105344

Custom High Fat/High Glucose Diet (#103806)
with 1% Matcha

Ingredient	kcal./gm	grams/kg	kcal./kg
High Nitrogen Casein	3.58	200	716
L-Cystine	4	3	12
Sucrose	4	90	360
Cornstarch	3.6	0	0.000
Dyetrose	3.8	50	190
Dextrose	3.64	300.562	1094.0
Soybean Oil	9	70	630
t-Butylhydroquinone	0	0.014	0
Whole Butter (18% Water)	9	181	1629
Ethoxyquin	0	0.024	0
Lard	9	20	180
Cellulose	0	50	0
Mineral Mix #210025	0.88	35	30.8
Vitamin Mix #310025	3.87	10	38.7
Supplement #410750	3.8	10	38
Choline Chloride	0	1.4	0
Matcha (provided)	0	10	0
(roughly 31 grams of water will evaporate)		**1031.000**	**4918.546**

https://doi.org/10.3390/nu18182961

2. Supplementary results

2.1 Metabolic phenotype of mice

Detail metabolic phenotype of mice during 10-week dietary intervention is provided in the figure S1 below. Overall, all mice under high-fat high-sugar diet supplementation including matcha group continuously gain body weight for 10-week period and body weight gain followed the same trend. WAT, serum cholesterol also confirmed successful induction of obesity by high fat diet and intervention did not showed significant intervention compared to HFD.

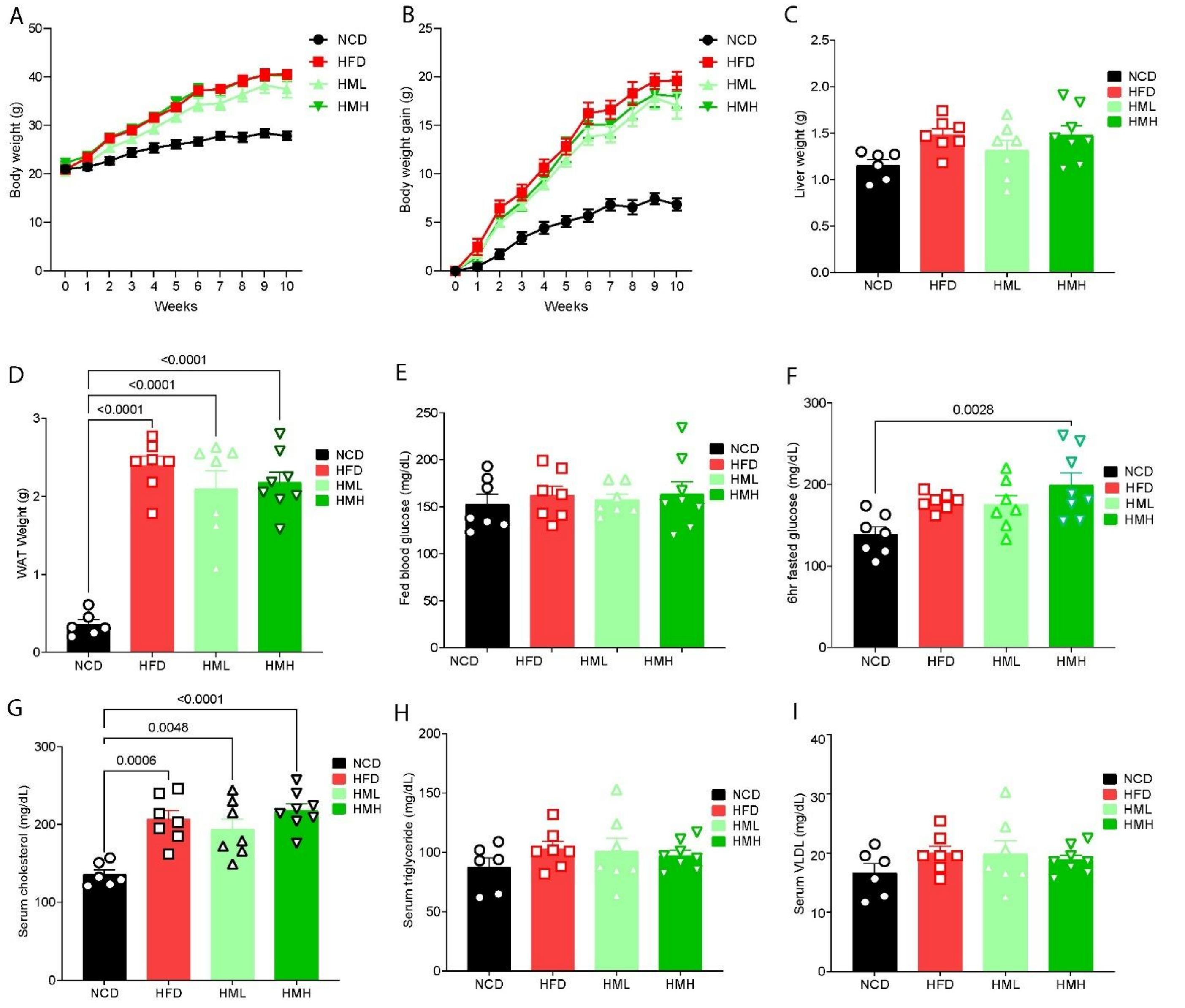

Figure S1: Body weight (A), body weight gain (B), liver weight (C), WAT weight (D), fed blood glucose level (E), 6hr fasted blood glucose level (F), serum cholesterol level (G) serum triglyceride levels (H) and serum VLDL level (I) of mice under 10-week matcha supplementation. Data are presented as the mean ± SEM (n = 6–8 mice per group). Statistical significance was determined by one-way ANOVA followed by multiple-comparison test. P < 0.05 was considered sta-tistically significant. (NCD: normal chow diet; HFD: high fat high sugar diet; HML: high fat high sugar diet with 0.2% matcha; HMH: high fat high sugar diet with 1% matcha; WAT: epidydimal white adipose tissue).

2.2 Hepatic triglyceride and cholesterol

Hepatic triglyceride, cholesterol in liver of mice after 10-weeks dietary intervention is provided in figure S2 below. Overall hepatic triglyceride was significantly increased in both HFD, HML, HMH group compared to NCD group. Matcha at both dose did not improve hepatic triglyceride deposition.

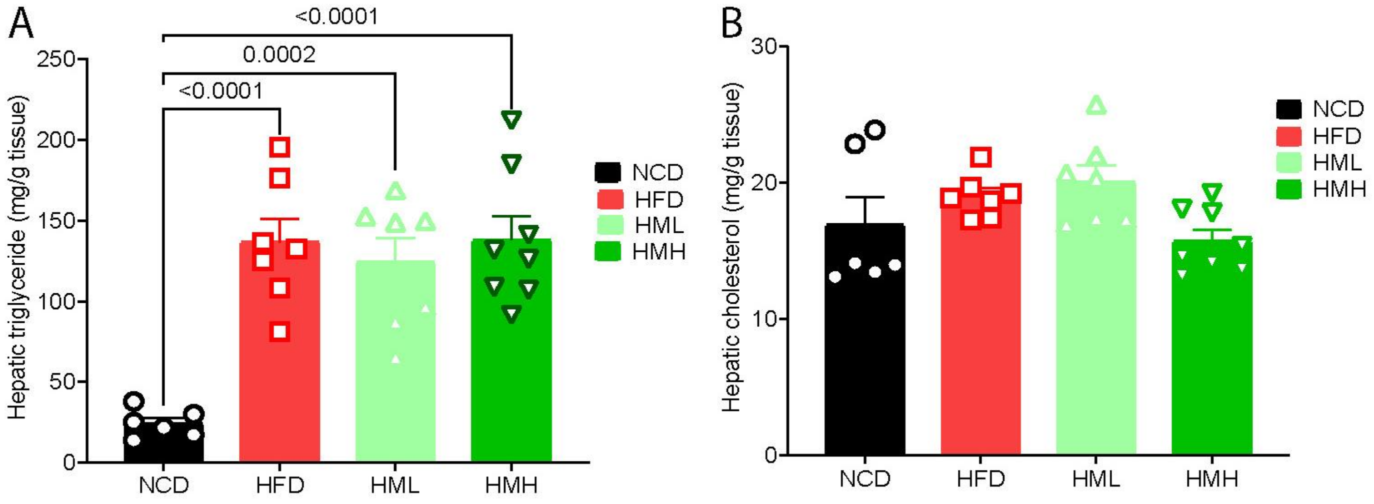

Figure S2: Hepatic triglyceride (A), and hepatic cholesterol (B) of mice under 10-week matcha supplementation. Data are presented as the mean ± SEM (n = 6–8 mice per group). Statistical significance was determined by one-way ANOVA followed by multiple-comparison test. P < 0.05 was considered sta-tistically significant. (NCD: normal chow diet; HFD: high fat high sugar diet; HML: high fat high sugar diet with 0.2% matcha; HMH: high fat high sugar diet with 1% matcha).

2.3. H and E staining and measurement of ventricular thickness and area

H and E staining images and quantification results for LV wall thickness and LV chamber area is presented in Figure S3 below. Overall, We observed significantly less LV wall thickness in heart of HML and HMH mice compared to HFD cntrol. However, there was no significant change in LV chamber area due to treatment.

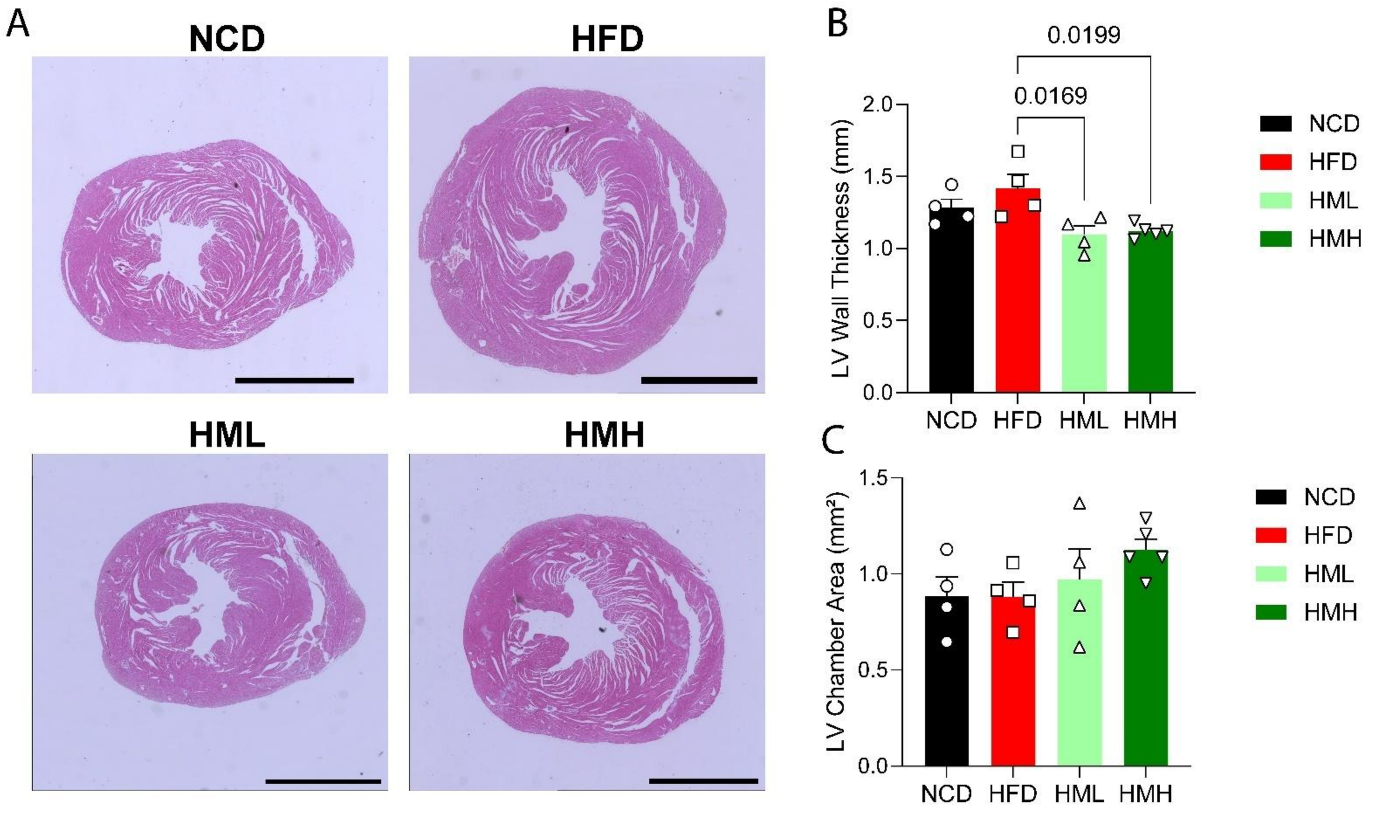

Figure S3: H and E staining images of heart under different treatment condition (A), LV wall thickness in mm for different treatment group (B) and LV chamber area in mm^2 for heart under different treatment condition (C). Statistical significance was determined by one-way ANOVA followed by multiple-comparison test. P < 0.05 was considered sta-tistically significant. (NCD: normal chow diet; HFD: high fat high sugar diet; HML: high fat high sugar diet with 0.2% matcha; HMH: high fat high sugar diet with 1% matcha). N=4-5 per group. Scale bar=2mm

2.4 Multivariate dispersion of microbiota for beta diversity analysis

Table S5: Analysis of multivariate dispersion of microbiota during beta diversity analysis

**Unweighted UniFrac**						
	Df	Sum Sq	Mean Sq	F	N.Perm	*P*
Groups	3	0.006852	0.002284	0.172	999	0.918
Residuals	18	0.239028	0.01327			
Weighted UniFrac						
	Df	Sum Sq	Mean Sq	F	N.Perm	*P*
Groups	3	0.007115	0.0023717	0.6673	999	0.619
Residuals	18	0.063979	0.0035544			

1.4

https://doi.org/10.3390/nu18182961

2.5 Pairwise comparision for beta diversity analysis with FDR adjustement

Table S6: Pairwise PERMANOVA comparisons of gut microbiota community based on weighted and unweighted UniFrac distance matrices. Gray-shaded cells indicate comparisons based on unweighted UniFrac distances.

	NCD	HFD	HML	HMH
NCD		R^2: 0.2445<br>p.adj: 0.1139	R^2: 0.1025<br>p.adj: 0.4595	R^2: 0.4244<br>p.adj: 0.0140
HFD	R^2: 0.5271<br>p.adj: 0.0090		R^2: 0.2761<br>p.adj: 0.0509	R^2: 0.4706<br>p.adj: 0.0140
HML	R^2: 0.5272<br>p.adj: 0.0090	R^2: 0.2958<br>p.adj: 0.0090		R^2: 0.4802<br>p.adj: 0.0140
HMH	R^2: 0.5322<br>p.adj: 0.0090	R^2: 0.2071<br>p.adj: 0.0090	R^2: 0.3363<br>p.adj: 0.0090	

Supplementary references

1. Lamichhane G, Lee DY, Franks R, Olawale F, Jin JB, Egan JM, Kim Y. Curcumin-rich diet mitigates non-alcoholic fatty liver disease (NAFLD) by attenuating fat accumulation and improving insulin sensitivity in aged female mice under nutritional stress. *Biology*. 2024 Jun 26;13(7):472.

https://doi.org/10.3390/nu18182961
